# Difficulties in implementing best standards in transgender mental health care in different European countries

**DOI:** 10.1192/j.eurpsy.2025.138

**Published:** 2025-08-26

**Authors:** K. Başar

**Affiliations:** Psychiatry, Hacettepe University, Ankara, Türkiye

## Abstract

**Abstract:**

For decades, the standards of gender-related healthcare provided to trans and gender-diverse people have been prepared by international professional associations. These standards are often expected to rely on research evidence, but when the research evidence is not conclusive, an expert consensus is sought. In addition to the standards and guidelines updated with intervals, such as the Standards of Care for the Health of Transgender and Gender Diverse People, Version 8, and the Endocrine Treatment of Gender-Dysphoric/Gender-Incongruent Persons: An Endocrine Society Clinical Practice Guideline of the Endocrine Society, there are several national guidelines developed by the professionals in some European countries. There are significant similarities among these care principles concerning practical recommendations. However, the practice may vary depending on the differences in healthcare delivery systems and, more importantly, legal regulations. In general, international guidelines provide room for flexibility in practical applications. Yet, in recent years, there has been a significant change in many European countries with respect to the public appreciation of gender diversity and gender-related healthcare, mainly resulting from a politicized debate triggered by more conservative arguments rather than evidence. This backlash, which has also had a profound impact on the medical professional sphere, is a cause for concern. In this climate, it is becoming more challenging to develop and implement the standards of care for trans and gender-diverse individuals in many countries. Similarities of challenges in the implementation of standards also exist in the means of handling these challenges.

**Disclosure of Interest:**

None Declared

